# Usefulness of random-start progestin-primed ovarian stimulation for fertility preservation

**DOI:** 10.1186/s13048-021-00935-5

**Published:** 2022-01-04

**Authors:** Haipeng Huang, Yukiko Itaya, Kouki Samejima, Shunichiro Ichinose, Tatsuya Narita, Shigetaka Matsunaga, Masahiro Saitoh, Yasushi Takai

**Affiliations:** Department of Obstetrics and Gynecology, Saitama Medical Center, Saitama Medical University, 1981 Kamoda, Kawagoe, Saitama, 350-3550 Japan

**Keywords:** Breast cancer, Dydrogesterone, Fertility preservation, Letrozole, Ovarian stimulation

## Abstract

**Background:**

Progestin-primed ovarian stimulation (PPOS) has been used in infertility cases in recent years, and several reports have stated that it has oocyte collection results similar to those of gonadotropin-releasing hormone antagonist (GnRH-ant) protocol. For emergency fertility preservation, random-start ovarian stimulation is usually recommended. Therefore we compared the clinical outcomes of random-start PPOS with those of conventional random-start GnRH-ant protocols in fertility-preserving cases.

**Methods:**

We retrospectively examined 86 cycles of oocyte collection, of which 56 were random-start GnRH-ant and 30 were random-start PPOS for fertility preservation at our hospital between January 2016 and April 2021. The primary outcome was the number of mature oocytes per cycle. The secondary outcome was the number of vitrified blastocysts per cycle for embryo freezing cases.

**Results:**

No significant differences were noted in the number of days of stimulation, total dose of gonadotropin preparation, and the number of mature oocytes and vitrified blastocysts. The number of hospital visits for monitoring was significantly lower in the PPOS group. The start of menstruation before oocyte collection was significantly less in the PPOS group.

**Conclusions:**

Random-start PPOS and GnRH-ant were similar in oocyte collection results. PPOS can reduce the number of hospital visits, thus reducing patient stress. PPOS at the start of the luteal phase can prevent the start of menstruation during ovarian stimulation.

## Background

In fertility-preserving treatment, oocytes should be collected within 2 to 3 weeks after the patient examination to avoid delaying the start of treatment for the underlying disease [1]. Therefore it is better to start ovarian stimulation as soon as possible unless its yield is compromised.

Mounting evidence indicates that follicle development may occur 2 to 3 times in one menstrual cycle [2], and in 2013, Cakmak et al. reported the efficacy of a random-start gonadotropin releasing-hormone antagonist (GnRH-ant) protocol [3], which became the common ovarian stimulation technique in fertility-preserving cases. In a systematic review in 2021 [4], a conventional method that started stimulation from the early follicular phase (*n* = 1012) was compared with a random-start method that started stimulation from the late follicular and luteal phases (*n* = 641). Although not significantly different, the random-start method had a longer stimulation period (10.4 vs. 10.1 days, respectively) and higher total gonadotropin usage (2688 vs. 2575 IU, respectively) on average than those of the conventional method. Furthermore, although the number of mature oocytes collected was similar, the estradiol (E2) levels at the time of trigger were significantly lower in the random-start method on average than in the conventional method (1128 vs. 1855 pg/mL, respectively), reconfirming the safety of its use in fertility-preserving cases.

In contrast, progestin-primed ovarian stimulation (PPOS) has been used in infertility cases in recent years, and it was shown to prevent unexpected increases in luteinizing hormone (LH) more efficiently than GnRH-ant. PPOS is also more affordable, and several reports have stated that it has oocyte collection results similar to that of GnRH-ant [4, 5]. We believe that PPOS is suitable for the suppression of unexpected LH surges as a random-start method for fertility-preserving cases with sustained gonadotropin suppression due to negative feedback from progestins. Therefore, we conducted a comparative study between the random-start PPOS and GnRH-ant methods. This study is the first to show the usefulness of random-start PPOS in fertility-preserving cases.

## Materials and method

### Study setting and patients

We retrospectively examined 86 cycles of oocyte collection for fertility preservation at our hospital between January 2016 and April 2021. Breast cancer cases accounted for 73% of the cases, followed by hematological disorders at 15%. For patients with male partners either with or without legal marriage, oocyte and/or embryo cryopreservation were offered for fertility preservation. For patients without male partners, only oocyte cryopreservation was offered. Semen analysis for male partners was always performed before oocyte collection in couples with no history of pregnancy. The cases in this study did not have severe male infertility. The endpoints were the number of days of stimulation, total dose of gonadotropin, serum E2 concentrations on the day of ovulation trigger, oocyte maturation rate, fertilization rate in cases of embryo freezing, and good blastocyst rate. This research protocol was approved by the Saitama Medical Center, Saitama Medical University Ethics Review Board (approval number: 2575). This study was not funded, and it was performed retrospectively using electronic medical record information and ultrasound findings.

### Controlled ovarian stimulation

For referred fertility-preserving cases, ovarian stimulation was promptly started regardless of the menstrual cycle if oocyte collection was desired after counseling. A random-start PPOS or GnRH-ant protocol was used depending on the practice of the attending physicians and the patient preference of ovarian stimulation method.

Both methods were initiated by subcutaneous injection of recombinant FSH (GONAL-f; Merck). All of the breast cancer patients were referred to our hospital along with the information of estrogen receptor status which had been tested either on the biopsy sample or on the surgical specimen. For estrogen receptor-positive breast cancer cases, 5 mg/day of letrozole was used in combination from the start of ovarian stimulation to the day of trigger.

**Fig. 1:**
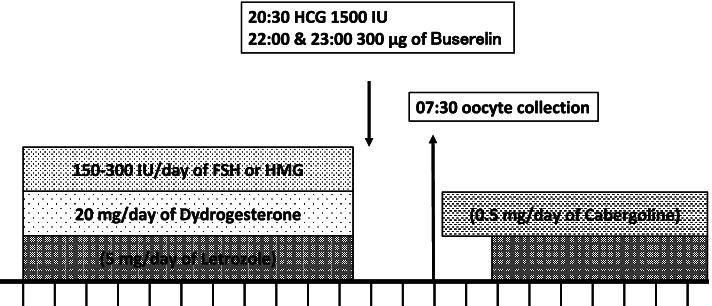
Random-start progestin-primed ovarian stimulation regimens. Abbreviations: FSH, follicle-stimulating hormone; HMG, human menopausal gonadotropin; HCG, human chorionic gonadotropin

In the GnRH-ant group, a flexible method was adopted wherein 0.25 mg of ganirelix acetate (Ganirest, MSD) was administered daily from the day when the main follicle diameter reached 14 mm by ovarian stimulation. Follicular diameter was defined as the average value of the major and minor axes. In contrast, in the PPOS group, 20 mg of dydrogesterone (Duphaston, Mylan) was administered each day from the start of ovarian stimulation to the day of trigger (Fig. 1). In both groups, follicular growth was monitored every 2–5 days using serum levels of FSH, LH, E2, and progesterone (P4), as well as transvaginal ultrasonography.

In both groups without letrozole, in general, when it was confirmed that there were ≥ 2 follicles of ≥18-mm diameter, dual trigger with four nasal sprays (150 μg each) of buserelin (Buserelin, Fuji) and an injection of 1500 IU of urine human chorionic gonadotropin (HCG) (HCG, Fuji) was administered to induce maturation of oocytes, and oocyte pickup was performed 35–37 h later. In the groups with letrozole, however, the dual trigger was delayed until when there were ≥ 1 follicles of ≥20-mm diameter. Also, even when the sizes of dominant follicles reach 18 or 20 mm, the time of triggering may be slightly delayed when the sizes of other nondominant follicles do not reach 16 mm in order to maximize the number of maturate oocytes.

### Embryo culture and assessment

Patients with male partners who opted for embryo cryopreservation underwent intracytoplasmic sperm injection (ICSI), in vitro fertilization (IVF), or split insemination after oocyte collection. Although selection was based mainly on the preference of the patient, ICSI was actively recommended in cases with poor semen findings on the day of oocyte collection. The cases in this study did not include severe male infertility.

### ICSI

On day 0 of oocyte retrieval, the collected eggs were pipetted to remove the granulosa cell layer (PINU06-20FT, Prime Tech Ltd.) connected to a piezo-electric actuator (Prime Tech Ltd.). After injecting sperm, the cells were cultured using a single-step culture medium (SAGE 1-Step, CooperSurgical). Embryos were observed in the time-lapse culture system (Astec Co. Ltd.) on days 1, 2, 3, 5, and 6 after oocyte retrieval. Normal fertilization was determined if two pronuclei were observed on day 1 after oocyte retrieval. Blastocysts were evaluated according to the blastocyst scoring system developed by Gardner et al. [6], and blastocysts that reached Grade 3BB were frozen.

### IVF

Within 2 h of oocyte collection, the final sperm concentration was adjusted to 300,000/mL, and insemination was performed. Fertilization was determined when the granulosa cell layer was removed, and the second polar body was confirmed approximately 5 h after fertilization.

### Study outcomes

The primary outcome was numbers of oocytes and metaphase two oocytes per cycle. The secondary outcome was the number of vitrified blastocysts per cycle for embryo freezing cases. In addition, AMH levels, ovarian stimulation period, total dose of gonadotropin preparation, days of hospital visits, start of menstruation rate before oocyte collection, number of collected oocytes, number of mature oocytes, and number of normal fertilized oocytes were comparatively examined. Oocyte maturation was evaluated at the timing of oocyte vitrification and ICSI. For the oocytes with which standard IVF was performed, oocyte maturation was evaluated under the removal of cumulus cells 4 h after insemination.

### Statistical analysis

All statistical analyses were performed using JMP version 16.0.0 software. Patient characteristics, ovarian stimulation characteristics, and endocrinological characteristics were expressed as mean (± standard deviation). For frequency/ratio comparisons, chi-squared test with Yates’ correction or Fisher’s exact test and the average ratio were used. The Student’s t-test was used for comparison. Statistical significance was set at *P* <  0.05.

## Results

### Patient characteristics

Table 1 compares the characteristics of the patients. There were no significant differences in age, history of pregnancy, the presence or absence of male partner, BMI, serum AMH levels, and the timing of ovarian stimulation (follicular or luteal phase). There were no patient with history of infertility or polycystic ovary syndrome in the present study.

**Table 1:** Comparison of patient characteristics between random-start GnRH-ant and random-start PPOS groups

	GnRH-ant, *n* = 56	PPOS, *n* = 30	*P* value
Age, years	32.6 (0.9)	31.7 (1.2)	0.531
Primigravida, %	75.0%	73.3%	0.865
No male partner, %	66.1%	53.4%	0.256
BMI	21.8 (0.5)	20.9 (0.6)	0.288
AMH, ng/mL	2.76 (0.37)	3.47 (0.51)	0.268
Luteal phase start, %	41.8	22.6	0.099

Values are expressed as the mean (± standard deviation)

### Outcomes of ovarian stimulation

Table 2 shows the outcomes of ovarian stimulation in both groups. The total amount of gonadotropin used, administration periods, number of eggs collected, and maturation rates of eggs were similar. No cases were canceled due to unexpected side effects in either group. The start of menstruation before oocyte collection was significantly lower in the PPOS group than in the GnRH-ant group (3.3% vs. 25.0%, respectively; *P* = 0.011). The number of hospital visits for monitoring was significantly lower in the PPOS group than in the GnRH-ant group (3.4 vs. 4.1, respectively; *P* <  0.001). When the comparison of visits was performed separately for the follicular-phase stimulation cases and for the luteal-phase stimulation ones, the number of visits in PPOS was still significantly lower than that in GnRH-ant (data not shown).

**Table 2:** Comparison of oocyte collection results

	GnRH-ant, *n* = 56 (Letrozole combination, *n* = 38)	PPOS, *n* = 30 (Letrozole combination, *n* = 18)	*P* value
HMG dose	2527.6 (73.3)	2512.9 (100.2)	0.905
HMG duration, days	11.0 (0.3)	11.2 (0.4)	0.616
Number of collected oocytes	9.9 (0.9)	12.2 (1.2)	0.137
Number of mature oocytes	8.4 (0.8)	10.8 (1.1)	0.099
Maturation rate, %	84.1 (2.4)	89.5 (3.3)	0.195
Number of visits for monitoring	4.2 (0.1)	3.4 (0.14)	< 0.001*
Start of menstruation before oocyte collection, %	25.0	3.3	0.013*

Values are expressed as the mean (± standard deviation). Significant *P* values are presented in bold and denoted with an asterix

*Abbreviations*: *GnRH-ant* Gonadotropin-releasing hormone antagonist, *HMG* Human menopausal gonadotropin, *LH* Luteinizing hormone, *P4* Progesterone, *PPOS* Progestin-primed ovarian stimulation

### Embryological outcomes

Thirty-seven patients in the GnRH-ant group and sixteen in the PPOS group chose to freeze blastocyst. A comparison of the results is presented in Table 3. The fertilization rates, number of frozen blastocysts, and blastocyst rates were similar.

**Table 3:** Comparison of frozen embryo cases

	GnRH-ant *n* = 37 (Letrozole combination, *n* = 27)	PPOS, *n* = 16 (Letrozole combination, *n* = 10)	*P* value
HMG dose	2552.7 (97.3)	2438.2 (148.1)	0.521
HMG duration, days	11.1 (0.3)	10.9 (0.5)	0.658
Number of collected oocytes	9.8 (1.0)	10.3 (1.5)	0.779
Maturation rate, %	89.0 (2.4)	90.1 (3.7)	0.807
ICSI rate, %	78.4	87.5	0.704
Fertilization rate, %	67.9 (4.0)	64.6 (6.1)	0.655
Number of frozen blastocysts	2.8 (0.5)	2.3 (0.7)	0.551
Good blastocyst rate, %	59.8 (5.4)	61.3 (8.1)	0.872

Values are expressed as the mean (± standard deviation)

*Abbreviations*: *GnRH-ant* Gonadotropin-releasing hormone antagonist, *HMG* Human menopausal gonadotropin, *LH* Luteinizing hormone, *P4* Progesterone, *PPOS* Progestin-primed ovarian stimulation

### Aromatase inhibitors

Thirty-eight patients were included in the GnRH-ant group and eighteen patients in the PPOS group. The letrozole combination was administered in estrogen receptor-positive breast cancer cases, and significantly suppressed serum E2 levels on trigger day, but the number of stimulation days, amount of gonadotropin used, number of eggs collected, and mature egg rates were not affected. In addition, the fertilization rates, number of frozen blastocysts, and blastocyst rates were similar (data not shown).

## Discussion

This study is the first to show the usefulness of random-start PPOS in fertility-preserving cases. We found that there was no difference in oocyte collection results between the random-start GnRH-ant and PPOS methods. In addition, since progestin was continuously administered in PPOS, random-start PPOS significantly suppressed the start of menstruation before oocyte collection compared with random-start GnRH-ant method.

Uterine bleeding can be a major problem in hematological disorder cases with thrombocytopenia, and also for other cancer patients without thrombocytopenia, it may be an advantage of random-start PPOS that the appearance of menstruation does not bother patients and caregivers during ovarian stimulation and oocyte collection. As shown in the present study, moreover, PPOS can reduce the number of hospital visits for monitoring since it was reported to be more efficient at preventing LH surge than GnRH-ant protocol [7] and it is enough to start monitoring 1 week after the start of PPOS, while in GnRH-ant protocol monitoring should be started earlier and be more frequent to prepare for unexpected LH rise. Thus PPOS may reduce patients’ stress and gain an advantage in the cost-effectiveness together with the low cost of progestins.

Since the first report by Kuang et al. in 2015 [8], PPOS has been expected to perform as well as the GnRH-ant method, and there is consensus that it is an affordable and useful ovarian stimulation method with a low cancelation rate. Previous studies on PPOS have compared the GnRH-ant protocol with PPOS [4, 5, 9–11], and the target patients were mainly normal responders.

The effect of progesterone on oocyte quality has also been investigated. In the random-start GnRH-ant method, 238 cases that underwent freeze-all strategy were divided into two groups based on P4 concentrations at the time of trigger (< 1.5 ng/mL and ≥ 1.5 ng/mL). There were no significant differences in embryonic aneuploidy [12]. There is also a report on preimplantation genetic testing for aneuploidies (PGT-A) of PPOS [5] where PGT-A was performed on 785 blastocysts obtained by an age-matched GnRH-ant method or PPOS at 37 years of age. The results revealed similar rates for euploid embryos in both groups. Within this context, this could become the basis for providing random-start PPOS to fertility-preserving cases without concerns.

Breast cancer is the most common disease for which assisted reproductive technology is performed for fertility preservation. For hormone-dependent tumors, it is ideal to use letrozole, an aromatase inhibitor, to avoid an increase in serum E2 concentrations [13]. However, a meta-analysis recently confirmed that menopausal hormone replacement therapy containing progestins such as (levo) norgestrel, norethisterone acetate, and medroxyprogesterone acetate increases the risk of breast cancer, compared to never users or estrogen-only users [14]. We assume that PPOS with dydrogesterone may be relatively safe because the above-mentioned meta-analysis showed that its use less than 5 years had been no effect on the risk of breast cancer, but the safety of PPOS for breast cancer patients should be carefully elucidated in the future studies. We decided the dosage of dydrogesterone according to the previous study [15], in which MPA 10 mg/d is too strong for LH suppression compared with dydrogesterone 20 mg/d. Since we sometimes decrease the dosage of dydrogesterone to 10 mg/d when the serum LH level is suppressed below 1.0 mIU/ml, we assume that the optimization or individualization of progestin dosage is necessary, especially for breast cancer cases.

The limitation of this study is that it was a retrospective study with a small number of cases, and that it was a single center study and that most participants had breast cancer. By accumulating random-start PPOS cases with hematological symptoms such as thrombocytopenia, we expect future studies to investigate the possibility of reducing the risk complications in oocyte collection during menstruation. We also believe that patients who have overcome cancer treatment would want to know the pregnancy results when using collected oocytes and frozen embryos.

Random-start PPOS is a simpler and more affordable ovarian stimulation method than random-start GnRH-ant. Both methods yielded similar oocyte collection results. Moreover, PPOS in luteal phase can prevent the start of menstruation during ovarian stimulation and is considered to be particularly useful in cases with thrombocytopenia.

## Data Availability

Not applicable.

## Abbreviations

AMH: Anti-Mullerian hormone
BMI: Body mass index
FSH: Follicle-stimulating hormone
GnRH-ant: Gonadotropin releasing-hormone antagonist
HCG: Human chorionic gonadotropin
ICSI: Intracytoplasmic sperm injection
IVF: In vitro fertilization
LH: Luteinizing hormone
PGT-A: Preimplantation genetic testing for aneuploidies
PPOS: Progestin-primed ovarian stimulation
